# Clinical and cost-effectiveness of adapted cognitive behaviour therapy for non-cardiac chest pain: a multicentre, randomised controlled trial

**DOI:** 10.1136/openhrt-2016-000582

**Published:** 2017-05-16

**Authors:** Peter Tyrer, Helen Tyrer, Richard Morriss, Michael Crawford, Sylvia Cooper, Min Yang, Boliang Guo, Roger T Mulder, Samuel Kemp, Barbara Barrett

**Affiliations:** 1 Centre of Psychiatry, Imperial College London, London, UK; 2 Department of Psychiatry and Applied Psychology, Institute of Mental Health, University of Nottingham, Nottingham, UK; 3 School of Public Health, Sichuan University, Chengdu, Sichuan, China; 4 Department of Psychological Medicine, University of Otago, Christchurch, New Zealand; 5 Respiratory Medicine, Royal Brompton and Harefield NHS Foundation Trust, London, UK; 6 Kings Health Economics, Kings College, London, UK

**Keywords:** non-cardiac chest pain, cardiac anxiety, cost-effectiveness, randomised controlled trial, health service use

## Abstract

**Objective:**

To investigate the cost-effectiveness of a modified form of cognitive behavioural therapy (CBT) for recurrent non-cardiac chest pain.

**Methods:**

We tested the effectiveness and cost-effectiveness of a modified form of CBT for chest pain (CBT-CP)(4–10 sessions) in patients who attended cardiology clinics or emergency medical services repeatedly. Patients were randomised using a remote web-based system to CBT-CP or to standard care in the clinic. Assessments were made at baseline and at 6 months and 12 months. The primary outcome was the change in the Health Anxiety Inventory Score at 6 months. Other clinical measures, social functioning, quality of life and costs of services were also recorded.

**Results:**

Sixty-eight patients were randomised with low attrition rates at 6 months and 12 months with 81% of all possible assessments completed at 6 months and 12 months. Although there were no significant group differences between any of the outcome measures at either 6 months or 12 months, patients receiving CBT-CP had between two and three times fewer hospital bed days, outpatient appointments, and A&E attendances than those allocated to standard care and total costs per patient were £1496.49 lower, though the differences in costs were not significant. There was a small non-significant gain in quality adjusted life years in those allocated to CBT-CP compared with standard care (0.76 vs 0.74).

**Conclusions:**

It is concluded that CBT-CP in the context of current hospital structures is not a viable treatment, but is worthy of further research as a potentially cost-effective treatment for non-cardiac chest pain.

**Trial registration number:**

ISRCTN 14711101.

**Key questions**

**What is already known about this subject?**

- We searched PubMed from 1 January 1996 to 31 December 2015, using the search terms ‘non-cardiac chest pain’, ‘non-specific chest pain *” and ‘atypical chest pain *”, with no language restrictions. We identified four systematic reviews from the same Cochrane group published between 2005 and 2015, the last being an update of all previous reviews.
- The 2015 review identified a total of 17 randomised controlled trials with 1006 randomised participants. Although the review suggested that psychological interventions, mainly using a cognitive-behavioural approach, were of some value in reducing chest pain and chest pain frequency, this was shown only in the first 3 months following the intervention and lost between 3 months and 12 months. Three of the studies were judged to have a high risk of outcome bias and there was high heterogeneity in the data. The authors concluded that there were ‘modest to moderate benefit for psychological interventions, particularly those using a cognitive-behavioural framework, which was largely restricted to the first 3 months after the intervention.’ They also suggested that further randomised controlled trials of psychological interventions for non-specific chest pain with follow-up periods of at least 12 months were needed.

**What does this study add?**

- By selecting a population that had at least two episodes of non-cardiac chest pain within the previous year we had a population that was less likely to remit spontaneously and have greater pathology. We also included a cost-effectiveness element, unlike any of the previous studies, and this showed that our treatment led to fewer outpatient appointments, A & E attendances, and fewer occupied bed days, accompanied by some cost savings. We extended assessment to 12 months after randomisation and although there were no significant group differences, the benefits at 12 months with cognitive behavioural therapy (CBT) compared with standard care were greater than at 6 months, unlike other trials. The lack of significant benefit was a consequence of the trial being underpowered, and we have concluded that the failure to recruit was for reasons that are linked to the current attitudes towards the management of chest pain in the hospital system, rather than the absence of suitable patients willing to take part. Further randomised trials are best carried out using a stepped care approach with a multidisciplinary team of psychological and medical personnel.

**How might this impact on clinical practice?**

- We conclude that there is little to be gained by further trials of this subject unless the structure of current services is changed. Before patients with non-cardiac chest pain are referred for CBT or any other psychological treatment there needs to be an integrated assessment involving psychological and physical components to ensure homogeneity and exclusion of those with transient or other non-cardiac symptomatology.

## Introduction

Non-cardiac chest pain has the status of a medical diagnosis in the 10th Revision of the International Classification of Diseases(ICD-10)(R07.89- non-cardiac chest pain) but shares this diagnosis with 20 other synonyms under the general heading of ‘other chest pain’ and is highly heterogeneous. It includes a range of disorders including a general description only (non-cardiac chest pain, atypical chest pain, non-specific chest pain), muscular tension (chest pain, musculoskeletal, chest pain, tightness, musculoskeletal chest pain), pain generated in other structures apart from the heart (sternal chest pain, chest wall pain, localised chest pain) and pain related to exertion (exertional chest pain, chest pain on exertion). None of the diagnostic labels include any indication of a psychological component, even though there is abundant literature on psychological interventions for the condition.^1–3^

Despite this lack of attribution, it is suspected that a substantial proportion of patients with non-cardiac chest pain have psychological causes, or at least can be treated appropriately using psychological means. Possible causes include excessive health anxiety (termed illness anxiety in the 5th Revision of the American Diagnostic Statistical Manual (DSM-5 4), one or more variants of somatic symptom disorder, the former diagnosis of hypochondriasis, and obsessive compulsive disorder. But because these diagnoses are given by mental health professionals, and not by the physicians who make the diagnosis of non-cardiac chest pain, they are seldom recorded in practice. Health anxiety has recently been associated with a future ischaemic heart disease in the general population over 12 years even when known risk factors for cardiovascular disease are controlled for.^5^ Health anxiety is also associated with adverse cardiac events in those who had myocardial infarction over the subsequent 4 years.^6^ Therefore health anxiety may be important to recognise and treat in its own right because of poor cardiac outcomes. Thus, despite good evidence that most of the people who present with non-cardiac chest pain do not have heart disease,^7 8^ we do not know how many have physical causes or precipitants and how many have psychological ones.

Previous work has suggested that many of these patients can be treated successfully in a relatively short number of sessions with different forms of cognitive behavioural therapy (CBT). Two findings gleaned from randomised trials are noteworthy. The effects of treatment are relatively modest, rapidly achieved but short-lasting (with maximal benefit at 3 months and usually lost by 6 months), and the number of patients who are potentially eligible for trial inclusion far exceeds those who actually take part.^9^ In planning a new trial on the basis of new evidence of a modified form of treatment^10^ we therefore wished to maximise the recruitment rate, have a longer period of follow-up, and to try and select patients with psychological causes for their chest pain.

## Method

The design of the COPIC (Cognitive therapy for Pain In the Chest) study was a simple two-treatment parallel design with equal allocation to either an adapted form of CBT for chest pain (CBT-CP) or to standard care in the relevant NHS services. The randomisation was carried out by an independent clinical trials unit (Health Services Unit, CHaRT, University of Aberdeen) with equal allocation to CBT and ST, with initial help given from Open-CDMS, a similar independent unit.

### Inclusion and exclusion criteria

Inclusion criteria were (1) significant chest pain on at least two separate occasions in the past year in which no significant pathology explaining the symptoms was found, (2) signed consent to take part in the study, (3) age between 18 years and 75 years. The exclusion criteria were (1) under active psychiatric care, (2) having received a prescription of a new psychoactive drug within the previous 2 months, (3) receiving, or on waiting list for, a formal psychological treatment. Those who were currently stable and on regular psychoactive medication (for more than 2 months) were eligible for the study. By seeing patients who had presented more than once in the past year it was hoped that a more resistant cohort would be recruited with less likelihood of non-specific improvement.

### Assessments

It was not clear which psychopathology would be most prominent in these patients, but because health anxiety had been found to be a common feature in many patients presenting to cardiology clinics,^10^ the change in scores on the short form of the Health Anxiety Inventory (HAI)^11^ was chosen as the primary outcome.

A more specific outcome, but not one that had previously been validated, was the Health Anxiety Questionnaire^12^ adapted for Chest Pain (Lucock and Morley Health Anxiety Questionnaire – Chest Pain (LMHAQ-CP), which included special questions on chest pain agreed with the original authors. The original Health Anxiety Questionnaire was also being used as the primary outcome in a parallel study in Christchurch, New Zealand, and so it was felt important to be included. Other assessments included self-completed analogue ratings of both the frequency and severity of chest pain and discomfort developed with the aid of a patient (Inskip Scale)(Appendix), self-ratings of generalised anxiety and depression (using the Hospital Anxiety and Depression Scale (HADS Scale),^13^ and social functioning using the Social Functioning Questionnaire.^14^ The Schedule for Evaluating Persistent Symptoms (SEPS),^15^ that had been found in preliminary studies to be an accurate measure of medically unexplained symptoms, was also included. Quality of life was recorded using the Euroqol Quality of Life Scale (EQ-5D scale^16^) at 6 months and 1 year. In addition, all health service related costs were recorded using the Adult Service User Schedule^17^ in the 6 months before randomisation and at 6-month intervals subsequently until 1 year. Using these data the overall costs for patients in the two arms of the trial were compared as well as major cost items such as inpatient care.

### Target number of participants

From previous work with the HAI we calculated that a difference between the scores of 4 between two groups at follow-up is clinically significant. Using data from an unrelated randomised controlled trial of CBT in health anxiety^18^ we demonstrated a significant benefit between CBT and control with a sample of 49 patients. In this study with a standard deviation (SD) for the change of HAI at 1 year as 6.0 a sample size of 96 patients would have 90% power to demonstrate significance at the two-sided 5% significance level.

### Ethics and consent

All patients recruited to the study read a brief information sheet initially and randomly discussed with clinical staff involved in their care. Those that consented were then seen by a research assistant, who gave each person a full participant information sheet and explained the study. If, after full assessment and explanation, patients agreed to take part, a signed declaration of informed consent was completed.

### Procedure

Initially the trial was confined to Kings Mill Hospital, North Nottinghamshire but because of poor recruitment was subsequently extended to The Royal Berkshire Hospital in Reading and The Hillingdon Hospital in Middlesex after appropriate ethical amendments.

Patients satisfying the criteria for inclusion and lacking exclusion criteria who presented with chest pain to either cardiology clinics, acute medicine and/or accident and emergency departments at the three hospitals above were considered for the study. Two other centres were included by agreement but the lack of a local principal investigator prevented recruitment from ever starting. The procedures for the chest pain pathway are not the same at these three hospitals but are still likely to be representative of the UK as in most hospitals there is no standard pathway for the assessment of non-cardiac chest pain unless rapid access chest pain clinics are available.

### Avoidance of bias

Concern has been expressed that bias may have been present in previous trials because single-blind assessment was prevented by trial protocols.^9^ Because of this all assessors made assessments without any discussion of treatment and if for any reason allocation was disclosed at interview a different researcher was involved in future assessments.

### Randomisation

Patients identified as eligible for the trial by cardiology and accident and emergency staff, and willing to take part, were first assessed by an independent research assistant. After baseline assessment all ratings and demographic details are recorded on a secure online database, initially Open-CDMS (Open Source Clinical Data Management System) in London and from 2014, CHaRT (Centre for Healthcare Randomised Trials) in the Health Services Research Unit, University of Aberdeen. Patients were then allocated to either CBT-CP or standard care in permuted stacked blocks and stratified by study centre. The allocated treatment was then passed to the trial coordinator (SC) who, if the patient was allocated to CBT-CP, informed the next available therapist at the centre concerned and then the patient, GP and consultant. Patients allocated to standard care are informed by letter or phone call and the GP and hospital team also notified. Follow-up assessments were carried out by research assistants who were ignorant of the original allocation after 6 months and 12 months.

### Experimental interventions CBT-CP

This was given by nursing staff and psychologists trained and supervised by HT. The procedure initially was a modification of CBT for health anxiety (CBT-HA)^19^ and its essential features were linked to health anxiety, including a formulation made for a recent episode of chest pain, assessment of the behaviours that appeared to be maintaining the pain, building up a model of the cognitive theory of emotion with illustrations where necessary of the nature of symptoms and using the patient’s strengths in finding alternative strategies of dealing with chest pain. It was also felt important to check that any gains made were consolidated and, where they were not, to emphasise techniques of relapse prevention before finishing treatment, not least because of previous evidence that gains in other studies were not maintained.^9^ When it was found that health anxiety was not a prominent feature in the presentation the focus shifted to examining other underlying fears and worries and changing the format of sessions. These included anxieties about vulnerability to assault, extreme exhaustion after chest pain and the fear that some important pathology would be missed if assessment was not made urgently. These often only became apparent later in therapy. According to our planned protocol^20^ between 4 and 10 sessions were offered but some flexibility was allowed as in our previous study up to 15 sessions were needed in more complex cases.^10^

### Standard care

No change was made to the form of care given to the patients allocated to this group in either their primary or secondary care settings. Thus, at various times care involved appropriate testing, explanation of findings, reassurance and the opportunity for the patient to ask questions about the symptoms and the test results. These interventions were made by the relevant clinicians and not influenced by the trial investigators. GPs were informed about the allocation of each patient after randomisation but received no further information subsequently.

### Primary and secondary outcomes

The primary outcome was the reduction in scores on the HAI between baseline and 6 months. Although previous studies have recorded outcomes at 3 months we did not think all psychological treatment would have been completed by then so did not record at this time point. Secondary outcomes were (1) the reduction in visual analogue scores of frequency and intensity of chest pain, (2) reduction in LMHAQ-CP scores, (3) reduction in the total SEPS Score, (between baseline and 6 months and 1 year, (4) the number of attendances at Accident & Emergency Departments after 6 months and 1 year, (5) total health service costs in primary and secondary care at 6 months and 12 months, (6) reduction in generalised anxiety symptoms (on the HADS-Anxiety Scale after 6 months and 1 year, (7) reduction in depressive symptoms on the HADS-Depression Scale at the same time points and (8) change in mean LMHAQ-CP scores from baseline after 1 year.

### Statistical analysis

All analyses were by intention to treat, analysed as randomised. The primary analysis compared mean change scores of the HAI Scale from the baseline to 6 months between the treatment and control groups. Multilevel modelling (MLM)was used to estimate and test differences of mean change scores between the two comparison groups’ adjustment for baseline score.^21 22^ Centre influence on treatment effect estimates was initially controlled during modelling but results without adjusting centre influence were presented given the small patient samples.^23^ Missing values were imputed under missing at random assumption with REALCOME software.^24^ MLM was also performed with observed only data to check the robustness of treatment effect estimates sensitive to missingness. Analyses of secondary outcomes followed the same analytical procedure and statistical approaches. Stata V.14 was used for data analysis.

### Economic evaluation

The economic evaluation adopted a health and social care perspective by collecting all the service use data over the period of follow-up, and so allowing the total cost of the services used by each study participant to be summed. These costs were taken together with the cost of the CBT to generate the average costs for each randomised group. The costs were compared using standard t-tests, recommended for economic analysis as they allow for analysis of mean costs without transformation. The robustness of this approach was checked through the calculation of bootstrapped CIs.^25 26^ A limited cost-effectiveness analysis was also performed where the costs in the CBT-CP and ST groups were compared alongside the difference in quality adjusted life years (QALYs) derived from the EQ-5D scores.

## Results

**Figure 1:**
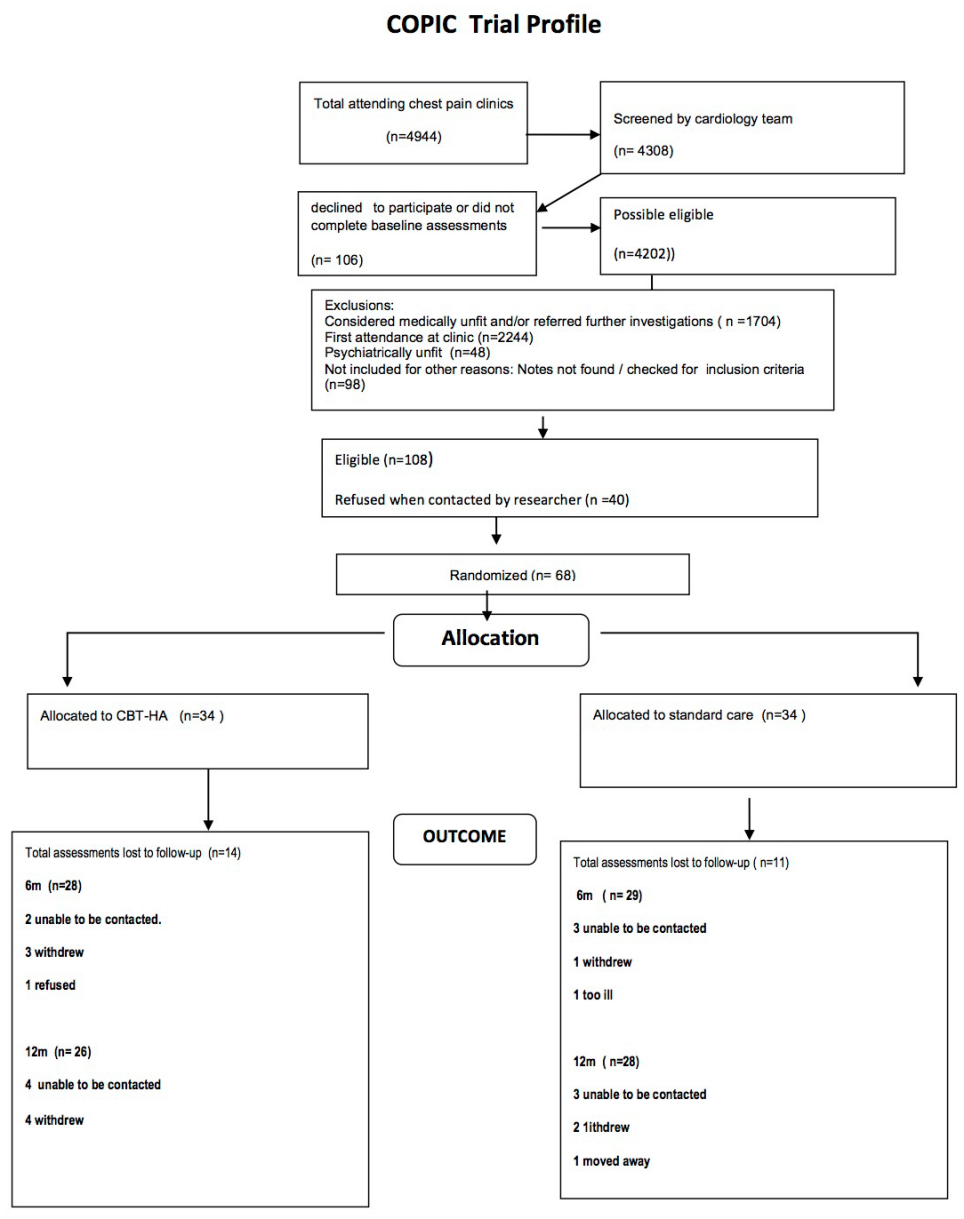
CONSORT diagram. CBT-HA, cognitive behavioural therapy for health anxiety.

Only 68 patients (47 M; 21 F) were recruited to the study in the 30 months between July 2012 and December 2014; this included a 9-month extension. As the study was underpowered greatly, significant group differences were neither expected nor found. Thirty-four patients each were randomised to the two groups. Attrition rates were low, with only 26 (19%) of the 136 possible follow-up points missing (figure 1). The mean number of treatment sessions in the CBT-CP group was 5.7 (range 0–15), carried out over a mean period of 14.3 weeks (SD 8.78). The longer period of treatment was sometimes necessary as there was considerable comorbidity. In the CBT-CP group 12 (35%) of the patients had other pathology (5 with previous cardiac disease independent of present symptomatology, 2 with gastrointestinal pathology probably linked to the chest pain (irritable bowel syndrome and gastric reflux), 2 with other psychopathology (chronic fatigue syndrome and post-traumatic stress disorder) and 3 with sexual health problems). One patient disclosed the nature of the allocation to the research worker during follow-up. Subsequently further assessments on this patient were made by an independent researcher.

The primary outcome was the change in HAI Scores at 6 months. This was probably an error in selection. Most patients (63%) did not have HAI Scores at or above the pathological level of 20 (table 1). At 6 months these scores showed greater improvement in the standard care arm (3.29, 95% CI −5.63 to –0.95) than the CBT-CP arm (2.35 95% CI −4.58 to –0.12), but the opposite effect was shown at 12 months (SC 4.21 95%CI −6.73  to –1.69; CBT-CP 4.97 95%CI−7.41 to –2.54).

**Table 1:** Baseline information

Variable	Standard Care (n=34)	CBT(n=34)
Age, years: mean(SD)	48.71 (13.46)	48.91 (14.50)
Centre: n(%)		
Notts	14 (41.8)	14 (41.8)
Hillingdon	7 (20.59)	6 (17.65)
Berks	13 (38.24)	14 (41.18)
Gender: n(%)		
Male	24 (70.59)	23 (67.65)
Female	10 (29.41)	11 (32.35)
Ethnicity: n(%)		
White British	29 (85.29)	24 (70.59)
Others	5 (14.71)	10 (29.41)
HAI: mean(SD)	16.29 (8.45)	18.13 (8.50)
HADS-Anx: mean(SD)	8.53 (4.81)	9.53 (4.43)
HADS0Dep: mean(SD)	5.01 (3.71)	6.12 (4.26)
SFQ: mean(SD)	4.56 (3.57)	5.16 (3.69)
EQ-5D: mean(SD)	7.79 (1.53)	7.03 (1.71)
HAQ-CP:mean(SD)	19.97 (12.80)	23.13 (12.53)
Pain frequency: mean(SD)	3.62 (1.46)	3.32 (1.87)
Pain severity: mean(SD)	5.41 (2.28)	6.24 (2.44)
SEPS symptoms: mean(SD)	5.79 (1.45)	5.85 (1.64)
SEPS mean: (SD)	14.97 (4.27)	16.21 (5.15)
No. of patients HAI≥20(%)	10 (29.41)	15 (44.12)

CBT, cognitive behavioural therapy; HADS, Hospital Anxiety and Depression Scale; HAI, Health Anxiety Inventory; HAQ-CP, Health Anxiety Questionnaire for Chest Pain; SEPS, Schedule for Evaluating Persistent Symptoms; SFQ, Social Functioning Questionnaire.

Although none of the treatment differences were significant (table 2) it was notable that for almost every measure there was greater improvement in the CBT-CP group than for standard care at 12 months compared with 6 months (table 2). The LMHAQ-CP showed the greatest differences between CBT-CP and standard care at 12m (mean difference 4.36, 95% CI −9.60 to 0.87, p=0.1).

**Table 2:** Results of multilevel modelling of change score (95% CI) from baseline and group difference (95% CI)

	Standard care (n=34) Mean change from baseline (95% CI)	CBT (n=34) Mean change from baseline (95% CI)	Group comparison Change difference (95% CI)	p value
HAI^*^				
6 months	−3.29 (**−**5.63 to to 0.95)	−2.35 (**−**4.58 to to 0.12)	0.94 (**−**2.30 to 4.17)	0.569
12 months	−4.21 (**−**6.73 to to 1.69)	−4.97 (**−**7.41 to to 2.54)	−0.76 (**−**4.11 to 2.59)	0.654
HADS_anx				
6 months	−2.39 (**−**3.80 to to 0.99)	−2.99 (**−**4.27 to 1.71)	−0.60 (**−**2.55 to 1.35)	0.546
12 months	−3.08 (**−**4.49 to to 1.67)	−4.15 (**−**5.65 to to 2.66)	−1.07 (**−**3.19 to 1.05)	0.319
HADS_dep				
6 months	−0.69 (**−**1.86 to 0.48)	−0.96 (**−**2.15 to 0.23)	−0.27 (**−**1.91 to 1.38)	0.75
12 months	−1.57 (**−**2.77 to 0.38)	−1.99 (**−**3.23 to 0.75)	−0.41 (**−**2.15 to 1.32)	0.639
SFQ				
6 months	−0.75 (**−**1.94 to 0.43)	−0.52 (**−**1.64 to 0.61)	0.24 (**−**1.39 to 1.86)	0.774
12 months	−0.22 (**−**1.42 to 0.98)	−0.94 (**−**2.17 to 0.29)	−0.72 (**−**2.55 to 1.11)	0.435
HAQ-CP				
6 months	−3.31 (**−**6.48 to 0.14)	−5.75 (**−**9.00 to 2.50)	−2.44 (**−**7.21 to 2.32)	0.313
12 months	−5.16 (**−**8.67 to 1.66)	−9.53 (**−**12.93 to 6.12)	−4.36 (**−**9.60 to 0.87)	0.101
INSKIP pain				
6 months	−1.08 (**−**1.64 to 0.52)	−0.43 (**−**0.97 to 0.10)	0.65 (**−**0.11 to 1.40)	0.095
12 months	−1.47 (**−**2.07 to 0.88)	−1.40 (**−**1.99 to 0.80)	0.08 (**−**0.71 to 0.87)	0.842
INSKIP severity				
6 months	−0.72 (**−**5.73 to 4.30)	−4.00 (**−**9.12 to 1.12)	−3.29 (**−**10.57 to 4.00)	0.376
12 months	−0.60 (**−**5.60 to 4.41)	−4.39 (**−**9.59 to 0.82)	−3.79 (**−**11.07 to 3.49)	0.307
Seps1symp				
6 months	−0.84 (**−**1.66 to to 0.02)	−0.38 (**−**1.12 to 0.37)	0.46 (**−**0.63 to 1.55)	0.407
12 months	−0.76 (**−**1.51 to 0.00)	−1.03 (**−**1.76 to to 0.30)	−0.27 (**−**1.30 to 0.76)	0.605
SEPS2 mean				
6 months	−2.76 (**−**4.69 to 0.82)	−2.93 (**−**5.01 to 0.85)	−0.17 (**−**2.83 to 2.49)	0.898
12 months	−3.80 (**−**5.85 to 1.76)	−4.69 (**−**6.97 to 2.41)	−0.88 (**−**3.94 to 2.17)	0.568

* Subgroup analysis of the high versus low HAI group (in table 1) showed no important differences between the four groups so identified and HAI scores were all lower at 12 months than at 6 months.

CBT, cognitive behavioural therapy; HAI, Health Anxiety Inventory; HADS-A, Anxiety section of Hospital Anxiety and Depression Scale; HADS-D, Depression section of Hospital Anxiety and Depression Scale; HAQ-CP, Health Anxiety Questionnaire for Chest Pain; SFQ, Social Functioning Questionnaire, INSKIP scale names after its inventor, Brian Inskip.

Pain measured in terms of frequency and severity by INSKIP scales, SEPS, Schedule for Evaluating Persistent Symptoms; SEPS1 (frequency of main symptoms), SEPS2 (disability produced by main symptoms).

### Economic analysis

Economic data were obtained on all patients at baseline, 85% at 6 months (85%) and 76% at 12 months follow-up. For full analysis 50 of the 68 patients were used (74%).

Over 12 months there was an observable difference in the use of hospital services between randomised groups; on average participants in the CBT group had fewer nights as inpatients and less than half as many outpatient appointments and Accident & Emergency (A&E) attendances as those in the standard care group (table 3). The use of community services was similar in both groups. The CBT-CP intervention cost on average was £480 per participant. The between-group differences in hospital service use is reflected in the cost of these services over follow-up. The CBT-CP group used on average £1771.52 less in hospital services, which more than covered the cost of the CBT-CP (table 4). Thus the average cost of all services over follow-up was £2235.53 in the CBT-CP group compared with £3732.02 in the standard care group. The difference was not statistically significant, largely because of a wide scatter of scores (p=0.798).

**Table 3:** Mean (SD) use of healthcare services by randomised groups over 12 months follow-up

	Standard (n=25)		CBT-CP (n=25)	
	Mean	(SD)	Mean	SD
Number of CBT sessions	0.00	(0.00)	5.72	3.49
Inpatient (nights)	3.20	7.16	1.12	3.05
Outpatient (appointments)	3.80	5.71	1.80	2.38
Day case (procedures)	0.12	0.33	0.32	0.69
Accident and emergency (attendances)	3.36	9.36	1.20	1.61
Diagnostic tests (number)	0.12	0.33	0.32	0.69
Community health and social care services (number)	6.40	17.03	3.76	6.64

CBT-CP, cognitive behavioural therapy for chest pain.

**Table 4:** Mean (SD) total cost (£) of service used over 12 months follow-up

	Standard (n=25)		CBT-CP (n=25)		Difference*	95% CI, p-value
	Mean	(SD)	Mean	SD		
CBT intervention	0	0	480.48	293.52		
Hospital costs	3403.48	6912.63	1631.96	2299.02		
Community costs	236.47	687.18	109.69	195.72		
Cardiac and psychotropic drug costs	44.75	96.37	87.20	137.60		
Total cost	3732.02	7346.85	2235.53	2434.86	−315.19	(−2782.69 to 2152.31), 0.798

* Adjusted for baseline cost.

CBT, cognitive behavioural therapy; CBT-CP, CBT for chest pain.

The utility scores, as measured using the EQ-5D were broadly similar and improved over time in both groups. There was a small and non-significant higher QALY over follow-up in the CBT group (0.76 QALYs) compared with the TAU group (0.74 QALYs) (table 5). Considering costs and outcomes together, the CBT-CP intervention resulted in lower overall costs and better outcomes.

**Table 5:** EQ-5D utility score and QALYs over 12 months follow-up

	Standard		CBT		Difference*	95% CI, p value
	Mean	(SD)	Mean	(SD)		
Utility baseline (n=68)	0.62	0.25	0.65	0.29		
Utility 6 months (n=56)	0.78	0.21	0.76	0.20		
Utility 12 months (n=52)	0.78	0.22	0.86	0.23		
QALY (n=52)	0.74	0.19	0.76	0.19	0.004	(−0.07 to 0.07), 0.900

CBT, cognitive behavioural therapy; QALY, quality adjusted life year.

## Discussion

The most unusual feature of this trial is that, despite addressing a very common problem in clinical practice, only 68 patients were recruited from three sites over a 30-month period. We are not in a position to determine why the recruitment rate was so low and are not sure how many eligible patients did not take part, but from the partial data we collected only about 20% of those who could have taken part, did so. In spite of this, those that did take part were adherent to treatment and had low levels of dropout, so it was clearly the recruitment uptake that was the main problem (accentuated by reluctance of cardiologists in three other centres to take part in the study despite ethical approval). As the trend for improvement was in favour of CBT-CP for almost all measures at 12 months, if the study had been adequately powered the results might have been different.

Our study represents the first trial of psychological treatment for non-cardiac chest pain to include a robust economic evaluation. The reduction in frequency of attendance at accident and emergency department, in outpatient clinics and in use of bed days, were notable in the CBT-CP group and illustrate the reduced demand on services that follows treatment. Between-group differences in costs were not significant, but the study was not designed with sufficient statistical power to detect them.

### Guidance for future research on this subject

During the course of this study it has been very apparent that the way in which services are currently configured do not allow psychological treatments to be adequately assessed. This is clear from previous studies; high levels of failure to recruit eligible patients and high dropout rates are prominent. A recent Cochrane review of psychological treatment for non-cardiac chest pain examined the effect of psychological interventions in a total of 17 randomised controlled trials involving 1006 participants. Despite these many studies the overall conclusion is tentative and of limited benefit to clinicians; ‘this review suggests a modest to moderate benefits of psychological interventions, particularly those using a cognitive behavioural framework, which was largely restricted to the first three months after the intervention’.^9^ Just over half of eligible patients agreed to take part and in the five largest studies, including 331 patients, the dropout rates ranged between 28% and 57%.

The previous studies have taken place over a period of more than 20 years and, not surprisingly, do not appear to have influenced practice except in a few centres. It is also of some concern that, when benefit has been shown, it is of such limited duration, and could be deemed not worthy of service adoption. The results of the COPIC Study should be seen in this context. Despite its inability to recruit an adequate number of patients, the results using CBT-CP suggest that the intervention was adding more than standard CBT, with economic benefits maintained over 1 year.

It is relevant that the supervisor of all the therapists in the study (HT) was a medically qualified doctor who had been employed in both primary and secondary care as well as having well developed skills in CBT. This allowed her to detect several other additional pathologies (oesophageal reflux, chronic fatigue syndrome, post-traumatic stress disorder), contributing to the non-cardiac chest pain, that would probably not have been detected by a psychologist or other therapist just trained in CBT. Five patients had previous cardiac pathology that had to be separated from current symptoms. These additional problems also complicated the length of the CBT-CP treatment so that most patients completed their treatment between 3 months and 6 months after randomisation. This may have been a factor in both the delayed improvement at 6 months and better outcomes at 12 months, so setting this study apart from previous ones.

This combined medical and psychological morbidity may have been more pronounced in the population of repeat attenders in the COPIC Study but is highly relevant both to continuing research in this area and to service provision. Future studies have to take into account several factors. Non-cardiac chest pain is inadequately treated at present and is associated with considerable morbidity, significant costs and poor outcomes.^27 28^ As the costs of care for physical disease are so much greater than those for the management of psychological problems, a marked saving could be made in a successful treatment programme.

In 1997 Mayou *et al* completed one of the early trials of CBT for non-cardiac chest pain in patients attending a cardiac clinic.^29^ Their findings are of interest because they adumbrated much of subsequent publication on the subject. After careful screening of 90 subjects they found 56 to be eligible but of these only 37 took part, with 60% completing all assessments at 12 months. This reluctance, according to the authors, ‘reflected the particular problems of introducing a psychological treatment to patients who had been referred to cardiologists with presumptive cardiac diagnoses’. This ‘abrupt change in clinical direction’ was clearly not acceptable to many patients. Mills and Mayou^30^ followed this subject up shortly afterwards in a series of editorials in this journal, and, while acknowledging that ‘it may well be difficult for cardiologists who are already hard pressed in providing cardiac assessments and investigations to consider spending more time on the issues covered in these editorials’, they pointed to the possible benefits, lying ‘in prevention of heart disease, routine clinical care, and the identification and treatment of disabling complications’.

Unfortunately, in the succeeding years, with the growth of rapid access chest pain clinics and much greater awareness of cardiac pathology fostered by the internet, psychological aspects have retreated a little, and it is likely that reluctance of both clinicians to offer psychological therapies and patients to accept them will increase unless there is a major change in thinking. Now that health anxiety and some other persisting types of anxiety have been shown to have adverse effects on cardiac outcome^5 6^ there is a need for a new approach involving integration of psychological assessment and treatment in cardiac teams such as those introduced by Chambers *et al* 31 to achieve better cardiac outcomes as well as address healthcare use in those with non-cardiac pain.

This might mean that patients are recruited to such studies in primary rather than secondary care, as in the primary care setting there is less pressure on practitioners to be absolutely sure they have excluded physical disease. Further studies in secondary care would be best focused on the group with the following inclusion criteria:-

- persistent or recurrent chest pain over a period of at least 6 months,
- repeated negative evidence of relevant physical pathology by examination and laboratory investigations (eg, negative troponin tests),
- psychological assessment by a combination of rating scales such as those used in this study^11–15^ as well as face-to-face assessment, to confirm psychopathology of anxiety, depression and somatic symptomatology.

Similar psychological assessments have been used in cancer services with good effect on outcomes.^32^ Better screening to firm up the exclusion of those who have other psychopathology is probably needed also.

Our findings, and the collective view of other studies, support comprehensively the notion that in secondary care, a multidisciplinary team including cardiologists, psychologists and nurses, preferably linked to rapid access chest pain clinics, should become a stepped care standard.^33^ It is not going to be satisfactory to refer patients for psychological treatment unless the mental sets and attitudes of the referring agencies are able to impart to the patient the likely benefits of a different approach to management, and without a strong medical component to the team other disorders such as oesophageal reflux may be wrongly referred for psychological input. In previous work we have found that general nurses can be highly effective in changing attitudes as well as providing treatment^34^ and when other members of the clinical team are all consistent in promoting biosocial alternatives for chest pain, adherence to treatment is likely to be much greater. In the linked study in Christchurch, New Zealand, much greater levels of recruitment were achieved as the project was nicely encapsulated as a ‘healthy heart’ intervention; this neatly joins up psychological and physical aetiologies.

What was also clear from the study was that the diagnosis of non-cardiac chest pain is a very unsatisfactory one as it is so heterogeneous, and this heterogeneity was emphasised by recruitment from both accident and emergency clinics and cardiology ones. This problem cannot be overcome by a simple screening procedure such as a questionnaire as no instrument would be able to disentangle the different causes. This heterogeneity probably reduced the efficacy of CBT-CP in reducing symptoms but the benefits in terms of reduced outpatient, inpatient and accident and emergency attendances, were nonetheless substantial. It also suggests that more general measures than specific symptoms, such as quality of life measured perhaps in greater depth than the EQ-5D such as the SF-6D (Short-Form Six-Dimension),^35^ might be a better choice of primary outcome. With the current demands for financial savings on all publicly funded health services, the economic benefits of introducing such a service are very persuasive. Common sense suggests a multidisciplinary medicopsychological team is better able to carry out this assessment than any other, and a large proportion of patients will need only very limited input to make a good recovery. The remainder could receive an intervention similar to CBT-CP to address the many complex factors that reinforce chest pain in vulnerable individuals.

## Conclusions

The combination of limited clinical improvement becoming amplified over time, together with possible economic gains, in this trial suggests that CBT-CP could be a cost-effective treatment for non-cardiac chest pain, but changes in service configuration, mental health literacy in hospitals and a different recruitment strategy are necessary to achieve its maximum benefit. An integrated service to assess both medical and mental pathology is considered necessary for progress to be made.
